# The D-amino acid oxidase inhibitor luvadaxistat improves mismatch negativity in patients with schizophrenia in a randomized trial

**DOI:** 10.1038/s41386-023-01560-0

**Published:** 2023-03-16

**Authors:** Patricio O’Donnell, Cheng Dong, Venkatesha Murthy, Mahnaz Asgharnejad, Xiaoming Du, Ann Summerfelt, Hong Lu, Lin Xu, Jens R. Wendland, Eduardo Dunayevich, Derek L. Buhl, Robert Litman, William P. Hetrick, L. Elliot Hong, Laura B. Rosen

**Affiliations:** 1 Takeda Pharmaceuticals USA, Inc, Cambridge, MA USA; 2 McLean Hospital, Department of Psychiatry, Harvard Medical School, Boston, MA USA; 3 Maryland Psychiatric Research Center, Department of Psychiatry, University of Maryland School of Medicine, Baltimore, MD USA; 4 Neurocrine Biosciences, Inc., San Diego, CA USA; 5 CBH Health, Gaithersburg, MD USA; 6 Department of Psychological and Brain Sciences, Indiana University, Bloomington, IN USA

**Keywords:** Schizophrenia, Predictive markers

## Abstract

Several attempts have been made to enhance N-methyl-D-aspartate (NMDA) receptor function in schizophrenia, but they have yielded mixed results. Luvadaxistat, a D-amino acid oxidase (DAAO) inhibitor that increases the glutamate co-agonist D-serine levels, is being developed for the treatment of cognitive impairment associated with schizophrenia. We conducted a biomarker study in patients, assessing several endpoints related to physiological outcomes of NMDA receptor modulation to determine whether luvadaxistat affects neural circuitry biomarkers relevant to NMDA receptor function and schizophrenia. This was a randomized, placebo-controlled, double-blind, two-period crossover phase 2a study assessing luvadaxistat 50 mg and 500 mg for 8 days in 31 patients with schizophrenia. There were no treatment effects of luvadaxistat at either dose in eyeblink conditioning, a cerebellar-dependent learning measure, compared with placebo. We observed a nominally significant improvement in mismatch negativity (MMN) and a statistical trend to improvement for auditory steady-state response at 40 Hz, in both cases with 50 mg, but not with 500 mg, compared with placebo. Although the data should be interpreted cautiously owing to the small sample size, they suggest that luvadaxistat can improve an illness-related circuitry biomarker at doses associated with partial DAAO inhibition. These results are consistent with 50 mg, but not higher doses, showing a signal of efficacy in cognitive endpoints in a larger phase 2, 12-week study conducted in parallel. Thus, MMN responses after a short treatment period may predict cognitive function improvement. MMN and ASSR should be considered as biomarkers in early trials addressing NMDA receptor hypofunction.

## Introduction

The N-methyl-D-aspartate (NMDA) hypofunction hypothesis of schizophrenia [1, 2] led to attempts to improve cognition and negative symptoms by enhancing NMDA receptor (NMDAR) function. Despite decades of efforts, the data so far have been inconclusive [3], with the NMDA hypofunction hypothesis being neither validated nor disproven. We recently discussed several challenges that may play a role in the lack of novel medications arising from this and other hypotheses, including the absence of reliable functional biomarkers that capture abnormal NMDA and circuitry-related function that could predict clinical efficacy [4].

A reasonable approach to enhance NMDAR function has been to elevate synaptic glutamate co-agonists, such as glycine and D-serine. These amino acids bind to a specific site in NMDAR [5], offering an opportunity to improve NMDAR function in a physiological manner. A potential role of D-serine in schizophrenia is supported by observations of reduced levels in plasma [6] and cerebrospinal fluid (CSF) [7] samples from patients. Furthermore, serine racemase, the enzyme that converts L-serine into D-serine, was identified as providing genetic risk for schizophrenia in genome-wide association studies [8]. Initial trials with the partial agonist D-cycloserine showed promising beneficial effects on negative symptoms [9–11]; however, subsequent studies failed to replicate these findings [12–14]. A meta-analysis revealed consistent, but small effect sizes for adjunctive NMDA modulation on negative symptoms [15]. D-serine administration reversed cognitive deficits and negative symptoms in patients with schizophrenia [16–18], but this effect required high doses [19]. It is possible that the optimal level of agonism at the co-agonist site of neuronal NMDAR cannot be achieved by exogenous amino acid administration, likely owing to peripheral metabolism before reaching the brain. Therefore, a more efficient approach to enhance NMDA function via increasing synaptic D-serine levels may be achieved by modulating metabolic enzymes, and the impact on cognitive deficits requires additional exploration.

D-serine is degraded by D-amino acid oxidase (DAAO) [20]. DAAO mRNA is highly expressed in the cerebellum [21], and DAAO protein has also been detected in the neocortex in postmortem studies [22], with higher expression in brains from schizophrenia patients than controls [23]. Blocking DAAO activity would increase synaptic D-serine levels, enhancing NMDAR function [24]. Add-on treatment with the weak DAAO inhibitor sodium benzoate improved a variety of symptom domains and cognitive function in patients with schizophrenia [25], indicating that DAAO inhibition is a promising approach.

Event-related potentials (ERPs) have emerged as reliable biomarkers to capture target engagement of glutamatergic drugs. Mismatch negativity (MMN) and auditory steady-state response (ASSR) stand out as endpoints impaired in schizophrenia [26–28] and sensitive to NMDAR pharmacology [29–31]. A potential for these measures to predict response to novel medications has not been demonstrated, as the studies have typically been underpowered or the agents that produced changes in ERPs did not improve clinical endpoints in the small studies conducted so far. It may be beneficial to evaluate the impact of novel drugs on ERPs and on clinical readouts in separate studies that are better suited to address them. Here we present data on a small study focusing on circuitry outcome measures that was run in parallel with a larger phase 2 study evaluating whether luvadaxistat improved negative symptoms and cognitive function in schizophrenia (INTERACT study; clinicaltrials.gov NCT03382639). While preliminary data revealed that luvadaxistat did not affect negative symptoms in that study, cognitive deficits were improved, offering an opportunity to compare ERP modulation with cognitive benefits.

Luvadaxistat (also known as TAK-831 and NBI-1065844) is a potent investigational DAAO inhibitor that was considered for the treatment of negative symptoms and is being developed for the treatment of cognitive impairment associated with schizophrenia (CIAS). Here, we evaluated the impact of luvadaxistat on NMDA- and cognition-associated biomarkers, as well as on cerebellar-circuitry-related biomarkers in patients with schizophrenia. Because DAAO has the highest expression in the cerebellum [21], the primary objective of the trial was to determine whether 8-day treatment with luvadaxistat improved learning assessed with eyeblink conditioning (EBC) compared with placebo. EBC requires cerebellar-circuitry integrity [32, 33], which is impaired in schizophrenia [34]. Given that D-serine treatment improved MMN in patients with schizophrenia [18], we also evaluated whether 8-day treatment with luvadaxistat improved MMN or ASSR at the gamma frequency band compared with placebo.

## Patients and methods

### Study design

This was a randomized, double-blind, placebo-controlled, two-sequence, two-period, crossover, phase 2a study assessing pharmacodynamic (PD) effects, safety, tolerability, and pharmacokinetics (PK) of multiple daily oral doses of luvadaxistat in adult patients with schizophrenia. The study was conducted in accordance with regulations relating to Good Clinical Practice, approved by the University of Maryland Baltimore Institutional Review Board, and registered at www.clinicaltrials.gov (NCT03359785). Written informed consent was obtained from all participants before enrollment. The study was sponsored by Takeda Pharmaceuticals and conducted at CBH Health in Gaithersburg, MD, USA, and the Maryland Psychiatric Research Center, University of Maryland School of Medicine, Catonsville, MD, USA, from January 10, 2018 to December 21, 2020.

**Fig. 1:**
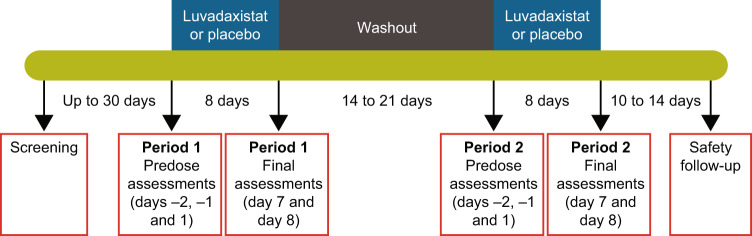
Study design. The study was a two-sequence, two-way crossover design in which patients were randomized to either 8 days of treatment with luvadaxistat 50 or 500 mg followed by 8 days of placebo, or 8 days of placebo followed by luvadaxistat 50 or 500 mg. Each treatment period was separated by a 2- to 3-week washout period. Assessments on day 7 or day 8 were flexible, with one day earlier or later allowed.

Patients were randomized to receive 8 days of luvadaxistat (either 50 mg or 500 mg once daily) followed by 8 days of placebo after a 2- to 3-week washout period, or 8 days of placebo followed by luvadaxistat 50 mg or 500 mg once daily after a similar washout (Fig. 1). The study started with 500 mg versus placebo and, after a planned interim analysis by an independent review body, the second part of the study was changed to 50 mg versus placebo, resulting in similar numbers of participants in both groups. The screening period (days −30 to −3) covered full medical, neurological, and psychiatric examinations. On day −2 of periods 1 and 2, participants were admitted to the clinic for 2-night stays for baseline clinical and cognitive assessment procedures and baseline electroencephalogram (EEG) and EBC assessments. In period 1, those who continued to meet all eligibility criteria were randomized via an interactive response technology system to one of two treatment sequences. On day 1, subjects underwent pre-dose blood PK/PD samples, first dosing, and post-dose blood draws, after which they were discharged from the clinic to continue dosing at their residence. Subjects received double-blind treatment from days 1 through 8 (inclusive) with completion of biomarker and PK/PD-related assessments on days 7 and 8 of each treatment period. Each patient served as their own control, with within-subject analyses planned a priori and active for each dose compared to the placebo data from those participants, not pooled placebo data.

### Participants

The study was conducted in 31 adult male and female subjects aged 18 to 60 years, inclusive, with a Diagnostic and Statistical Manual of Mental Disorders, Fifth Edition (DSM-5) diagnosis of schizophrenia, who were receiving stable antipsychotic therapy (no change in dose by >25% in the preceding 2 months). Subjects had a Positive and Negative Symptom Scale (PANSS) negative symptom factor score of at least 15, with minimal fluctuation between screening and baseline (<25% change). This was driven by the fact that negative symptoms were the primary indication being pursued at the time this study was designed. PANSS total score was not to exceed 90 points, with minimal fluctuation between screening and baseline (<20% change). Subjects with extrapyramidal signs or symptoms, or with depressive symptoms, were excluded.

### Pharmacokinetics and pharmacodynamics

Serial blood samples for PK analysis of luvadaxistat plasma concentrations and for D-serine and L-serine analyses were collected. Plasma concentrations of luvadaxistat, D-serine, and L-serine were measured by a validated high-performance liquid chromatography with tandem mass spectrometry.

### Eyeblink conditioning

Delay EBC was used to evaluate the impact of luvadaxistat on cerebellar-dependent learning. As described in greater detail elsewhere [33], EMG responses were recorded as blinks if the amplitude exceeded five standard deviations above the baseline (baseline window for each trial = 125 ms before CS onset). Conditioned responses were recorded if the blink occurred between 100 and 350 ms after CS onset. Trials with spontaneous blinks occurring within a window from 75 ms before CS presentation to 25 ms following CS onset were relegated as bad trials and excluded from further analysis.

### Event-related potentials

The event-related potentials (ERPs) measured (via scalp EEG) included MMN during the oddball paradigm, the ASSR task, and the P300 wave during an active oddball task assessing amplitude to target tone. ERPs were recorded using a 64-channel system (SynAmp 2; Compumedics Neuroscan) with sintered Ag/AgCl electrodes at 1 kHz sampling rate with bandpass at 0.1 to 200 Hz. Impedance was kept below 5 kΩ. Participants sat in a semi-reclining chair inside a sound-attenuated chamber. A midline frontal electrode (FZ) was used for MMN measurement because this location typically shows the largest patient-control differences on MMN [35, 36], and MMN was measured at a latency window that was selected a priori based on previous studies [37].

ASSRs were recorded in the same setting while participants listened to click trains, delivered by headphones at 2.5, 5, 10, 20, 40, and 80 Hz. Based on previous frequency-specific findings of ketamine effects and schizophrenia-related abnormalities [38, 39], we focused the analysis on gamma frequencies (40 Hz). An auditory screening test excluded apparent hearing impairment. The normalized ASSR power by stimulus frequency at gamma bands was the primary ASSR measure.

For P300 assessments, 600 auditory stimuli were presented, of which 80% were standard tones (60 ms, 1000 Hz) and 20% were target tones (60 ms, 1400 Hz) presented at 75 dB. All tones had a 5 ms rise/fall time, with a stimulus onset asynchrony of 1300 ms. Participants were instructed to press a response button as fast as they could whenever they heard the target tone. No response was required for the standard tone. P300 responses were measured at a midline parietal electrode (PZ) following the same eyeblink correction routine used for MMN. Records were filtered at 0.1 to 30 Hz in 24 dB/octave, epoched, baseline-corrected, and threshold-filtered at ±75 µV for artifact rejection. Standard and target trials were averaged separately, followed by peak detection within a 250 to 400 ms post-stimulus window and followed by visual inspection to verify correct placement of each peak. Scoring was blinded.

### Cognitive and clinical assessments

The Brief Assessment of Cognition in Schizophrenia (BACS) battery [40] was included in this study as an exploratory assessment to obtain estimates of potential effects of luvadaxistat on cognitive function. PANSS and other clinical assessments were also collected.

### Statistical analyses

The primary endpoint was the average percentage of conditioned responses in the EBC task at day 8. Pairwise comparisons between active treatments and placebo were generated with an analysis of variance (ANOVA) in a within-subject manner, with treatment sequence, period, and treatment as fixed effects. Subject nested within sequence was included as a random effect. The dependent variable was the change in response from the period baseline assessment to the final treatment day for each period, and the response data were logit transformed before the change was calculated. The primary endpoint was tested individually for each of the two doses against 5% (one-sided) level of significance. Sample size was determined based on prior studies using EBC and MMN in patients with schizophrenia. The secondary EEG endpoints were analyzed using an ANOVA model like that used for the primary endpoint and were tested individually for each of the two doses against 5% (one-sided) level of significance.

The changes from baseline in concentrations of D-serine, L-serine, and the ratio of D-serine to total serine were compared using a mixed model for repeated measures (MMRM). Sequence, period, treatment, and time point were fixed effects, and subject nested within sequence was a random effect.

## Results

### Demographics

In both luvadaxistat groups (500 mg and 50 mg), most participants were male (88.2% and 78.6%, respectively), Black or African American (94.1% and 71.4%, respectively), and not Hispanic/Latino (100% and 92.9%, respectively). The mean age was 34.7 years (range: 24 to 50 years), and the mean body mass index was 30.7 kg/m^2^ (range: 21 to 40 kg/m^2^) in the luvadaxistat 500 mg group, compared with 42.9 years (25 to 57 years) and 28.9 kg/m^2^ (24 to 40 kg/m^2^) in the luvadaxistat 50 mg group. Most participants in the luvadaxistat 500 mg and luvadaxistat 50 mg groups were current smokers (52.9% and 71.4%, respectively). Patient disposition is summarized in a CONSORT diagram (Fig. S1).

### Pharmacokinetics and pharmacodynamics

Mean plasma concentrations of luvadaxistat were higher for subjects treated with 500 mg than with 50 mg doses. No accumulation was observed for subjects receiving multiple doses of luvadaxistat 50 mg (mean plasma concentration [standard deviation (SD)] of 253.4 [199.3] ng/mL on day 1 [0.25 to 2.00 h] compared with 203.5 [199.6] ng/mL on day 7 [0.25 to 2.00 h]), and trough concentrations were low at day 7 (8.749 [19.212] ng/mL) and day 8 (3.366 [2.726] ng/mL). Some accumulation of luvadaxistat was observed in subjects treated with multiple daily doses of luvadaxistat 500 mg (mean plasma concentration [SD] of 700.4 [783.0] ng/mL on day 1 [0.25 to 2.00 h] compared with 1184 [482] ng/mL on day 7 [0.25 to 2.00 h]), and trough concentrations were higher than those observed following luvadaxistat 50 mg treatment (day 7: 78.92 [229.90] ng/mL; day 8: 21.61 [14.65] ng/mL).

Plasma D-serine levels increased following treatment with luvadaxistat 50 mg (range: 16.5% to 32.4%) and 500 mg (range: 11.3% to 36.4%). These levels were higher than those following placebo treatment (range: 3.2% to 7.8%). The MMRM analysis confirmed that D-serine levels following luvadaxistat treatment were significantly higher at post-baseline time points than those following placebo treatment (*p* < 0.05). Plasma L-serine levels were generally similar at all post-baseline time points following treatment with luvadaxistat 50 mg (range: 6.2% to 18.8%), 500 mg (range: −10.6% to 3.2%), or placebo (range: −8.5% to 5.5%). The ratio of D-serine to total plasma serine was greater at post-baseline time points following treatment with luvadaxistat 50 mg (mean: 14%; range: 6.8% to 21.8%) and 500 mg (mean: 39%; range: 9.6% to 60.5%) compared with placebo (range: 5.6% to 21.9%). Raw values are presented in Table S1.

### Safety and tolerability

Luvadaxistat was well tolerated, and all adverse events (AEs) were of mild intensity and not related to treatment. No serious AEs occurred. Clinical laboratory values were within normal limits for most subjects throughout the study. Three subjects following placebo treatment had a markedly abnormal laboratory value (bilirubin, creatinine, and glucose; one subject each [3.8%]), and one subject (7.1%) following luvadaxistat 500 mg treatment had a markedly abnormal creatinine value. The subject with the markedly abnormal creatinine value had a high pre-dose value of 3.15 mg/dL on day −2. The subject was brought back for a retest (unscheduled visit) 2 days later, and the creatinine value was normal (0.95 mg/dL). The subject also had other pre- and post-dose measurements that were within normal ranges; therefore, the abnormal value was not considered clinically significant.

### Luvadaxistat did not affect EBC

**Fig. 2:**
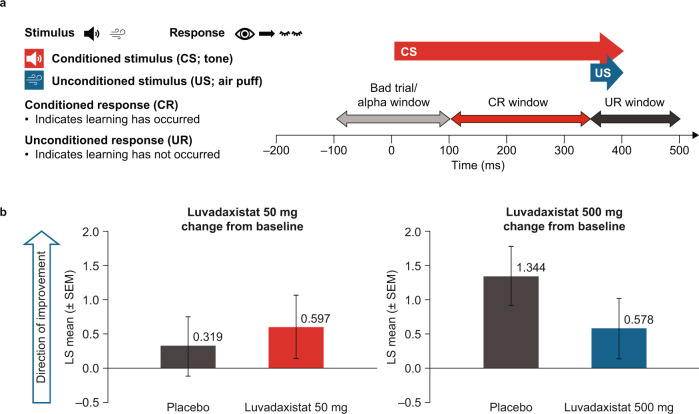
Eyeblink conditioning. **a** Eyeblink conditioning design includes a tone as the CS and an air puff as the US. As participants initially respond with an eyeblink to the US, learning mechanisms result in eyeblinks to the CS. We measured percentage responses to the CS before and after treatment. **b** Changes from baseline in percentage CR at Day 8 with luvadaxistat 50 mg compared with placebo (left) and with luvadaxistat 500 mg compared with placebo (right). CR conditioned response, CS conditioned stimulus, LS least-squares, SEM standard error of the mean, UR unconditioned response, US unconditioned stimulus.

The primary endpoint for this study was the average percentage of conditioned responses during the EBC test at day 8 as analyzed by ANOVA. The least-squares (LS) mean change from baseline in percentage conditioned response at day 8 was 0.597 ± 0.459 (estimate ± standard error of the mean [SEM]) for subjects treated with luvadaxistat 50 mg compared with 0.319 ± 0.432 for those treated with placebo and was 0.578 ± 0.444 for subjects treated with luvadaxistat 500 mg compared with 1.344 ± 0.428 for those treated with placebo (Fig. 2). These changes were not considered statistically significant for luvadaxistat 50 mg versus placebo (*p* = 0.2401) or luvadaxistat 500 mg versus placebo (*p* = 0.9053), and there was no statistically significant influence of sequence or period on the results. Thus, although there was a change in the hypothesized direction, luvadaxistat did not significantly affect EBC.

### Luvadaxistat reversed MMN deficits compared with placebo

**Fig. 3:**
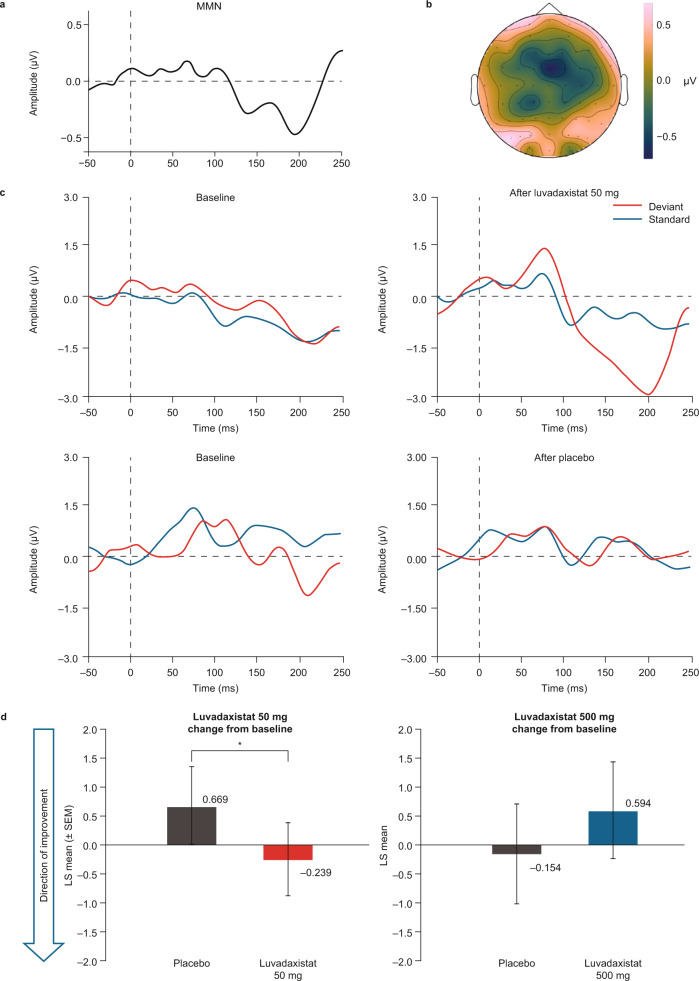
Mismatch negativity. **a** Difference waveform of grand averages of all recording, captured with subtracting deviant responses from standard responses. **b** MMN evoked potential distribution at the peak latency time of the MMN waveform in 3A. Amplitude is color-coded, and highest intensities were observed around frontocentral locations, where MMN amplitudes were measured for statistical comparisons. **c** Auditory evoked potential traces from a representative patient at baseline (left) and after treatment (right) for the luvadaxistat 50 mg sequence (top) and the placebo sequence (bottom). Blue lines illustrate the standard evoked potential, and red lines are the deviant (oddball) response. Time scale is in ms. The luvadaxistat 50 mg treatment, but not placebo, resulted in an increase in the deviant negative signal at approximately 150 ms, yielding an improved MMN signal in this patient that was not observed in the placebo period. **d** Bar graphs illustrating the group effects as changes from baseline in MMN amplitude at day 8 with placebo or luvadaxistat 50 mg (left) and with placebo or luvadaxistat 500 mg (right). The 50 mg treatment showed a nominally significant improvement in MMN compared with placebo. LS least-squares, MMN mismatch negativity, SEM standard error of the mean. *p = 0.0497.

MMN was a secondary endpoint in the study. Difference waves (Fig. 3a) and scalp distribution of the ERP signal (Fig. 3b) were in accordance with what is expected for MMN, with highest intensities around frontocentral locations, where MMN amplitude was measured. The LS mean change ± SEM from baseline in MMN amplitude at day 8 was −0.239 ± 0.363 for subjects treated with luvadaxistat 50 mg compared with 0.669 ± 0.382 for those treated with placebo and was 0.594 ± 0.492 for subjects treated with luvadaxistat 500 mg compared with −0.154 ± 0.501 for those treated with placebo (Fig. 3c, d). Raw MMN amplitude values are presented in Table S2. These changes were nominally statistically significant for luvadaxistat 50 mg versus placebo (*p* = 0.0497), with an effect size of −0.691, but not for luvadaxistat 500 mg versus placebo (*p* = 0.8517; effect size: 0.389). There was no statistically significant influence of sequence or period on the results. These results indicate that luvadaxistat had beneficial effects on a biomarker known to be altered in schizophrenia, but only at the lower dose tested.

### Luvadaxistat also affected ASSR

**Fig. 4:**
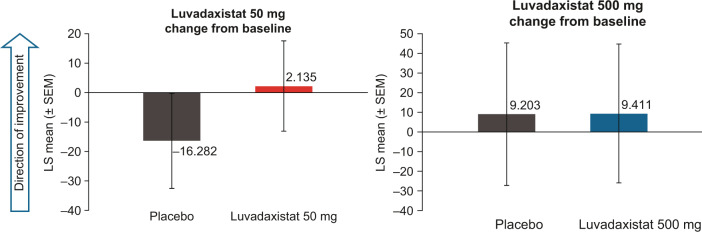
Auditory steady state. Bar graphs illustrating the changes from baseline in ASSR gamma band power at 40 Hz with placebo or luvadaxistat 50 mg treatment (left) and with placebo or luvadaxistat 500 mg treatment (right). ASSR auditory steady-state response, LS least-squares, SEM standard error of the mean.

ASSR gamma band power was another NMDA-related secondary endpoint in the study. The LS mean change ± SEM from baseline in ASSR gamma band power at day 8 was 2.135 ± 8.83 for subjects treated with luvadaxistat 50 mg QD compared with −16.282 ± 9.31 for those treated with placebo and was 9.411 ± 20.4 for subjects treated with luvadaxistat 500 mg QD compared with 9.203 ± 20.9 for those treated with placebo (Figs. 4, S2). These changes showed a statistical trend for luvadaxistat 50 mg (*p* = 0.0561), but not for luvadaxistat 500 mg versus placebo (*p* = 0.4954).

### P300 was unaffected by luvadaxistat

We also tested P300 ERP. The LS mean change ± SEM from baseline in P300 target amplitude at day 8 was −2.019 ± 1.68 for subjects treated with luvadaxistat 50 mg compared with 0.608 ± 1.68 for those treated with placebo and was 1.102 ± 1.77 for subjects treated with luvadaxistat 500 mg compared with 2.595 ± 1.86 for those treated with placebo (Fig. S3). These changes were not nominally statistically significant for luvadaxistat 50 mg versus placebo (*p* = 0.9185) or for luvadaxistat 500 mg versus placebo (*p* = 0.7193).

### Effects of luvadaxistat on cognition

We used the BACS to evaluate the impact of 8 days of luvadaxistat on cognition. All participants received the BACS assessments four times separated by 7 days. A learning effect with such repeated treatment combined with a higher-than-expected variability at baseline are important confounds that made the data uninterpretable. Therefore, we do not report the BACS data here.

## Discussion

Although it showed a trend to enhance but had no statistically significant effect on the primary endpoint, EBC, luvadaxistat significantly improved MMN and showed a trend toward improving ASSR, in both cases at 50 mg, but not with 500 mg. Luvadaxistat did not affect P300 or BACS at either dose. An important caveat in this study is the small sample size, so these results should be taken as exploratory in nature.

All changes in estimates in a favorable direction for MMN and ASSR occurred with the lower luvadaxistat 50 mg dose and were not observed with the higher 500 mg dose. This observation may be due to an inverted-U dose-response, and it is consistent with results from a parallel phase 2 study (INTERACT), in which 12 weeks of treatment with luvadaxistat 50 mg, but not luvadaxistat 500 mg, resulted in cognitive improvement [41]. Furthermore, an assessment of luvadaxistat’s effect on synaptic plasticity in mice also revealed an inverted-U dose-response [42], a profile not uncommon for glutamate pharmacology [43, 44]. Together, these studies suggest that the exposure and functional biomarker responses relationship of luvadaxistat is not linear. Although D-serine reduction in schizophrenia has been reported to be approximately 20% [6], the increases from baseline in the D-serine/total serine ratio were approximately 16% with luvadaxistat 50 mg and approximately 35% with luvadaxistat 500 mg, raising the possibility of overcorrection with the high dose. The mechanisms behind this profile remain unclear and need to be elucidated.

DAAO is highly expressed in the cerebellum, yet the main observation in this study is driven by MMN, a measure typically showing the largest response in the cerebral cortex. Given that we observed D-serine increases in plasma, and in CSF in a previous phase 1 study, it is possible that DAAO inhibition in the cerebellum yields enough increase in D-serine levels that reach NMDAR in other parts of the brain. Alternatively, an indirect impact of cerebellar function on MMN and other cortical ERPs cannot be ruled out [45]. A cerebellar role in schizophrenia has been proposed [46], and abnormal connectivity between cerebellum, thalamus, and cortex has been reported in schizophrenia [47]. In addition, there is evidence of DAAO function outside the cerebellum, given that luvadaxistat enhanced hippocampal LTP [42] and DAAO protein has been detected in the cortical region in human postmortem samples [22]. Thus, the specific neurocircuitry changes underlying the beneficial effect of luvadaxistat on MMN remain to be determined.

Because MMN and ASSR are strongly correlated with cognitive performance [29, 48], the beneficial effects observed here suggest that luvadaxistat could be beneficial in CIAS. Indeed, in the INTERACT study, while initial assessments revealed that 12 weeks of treatment with luvadaxistat in patients with schizophrenia did not meet the primary endpoint for improvement in negative symptoms, there was significant improvement in cognitive function assessed with the BACS [41]. The publication of the INTERACT study data is undergoing peer review, so the abstract should be considered preliminary. Notably, luvadaxistat showed a significant effect on MMN and a trend on ASSR in our study at the same dose that produced cognitive improvement in the larger phase 2 study following more extended treatment. The data from these two clinical studies provide the first indication that short-term DAAO-regulated glutamate pharmacology effects on MMN could be associated with cognitive improvement with longer dosing regimens.

There are some limitations with these results, however. The sample size was small, as recruitment was impacted by the COVID-19 pandemic. The placebo effects on MMN were different for the cohorts receiving 50 mg vs 500 mg. Yet, even with such small sample sizes there were nominally significant differences, so the data can be interpreted as reliable. Our use of one-sided p values is not standard and could be viewed as a limitation; however, this approach was set a priori as we hypothesized a MMN change in one direction. In addition, the difference in placebo effects between groups emphasizes the relevance and need of within subject comparisons with variable measures such as these ERPs.

A key learning from our study is that functional biomarkers should be used in early clinical development to de-risk later stages in drug development for psychiatry. The INTERACT study tested luvadaxistat 50 mg and 500 mg on the basis of what we knew about D-serine elevations from phase 1 studies, and 500 mg was hypothesized to be the effective dose because it was at the plateau of D-serine elevation, without our knowing the functional impact of such elevation. The current study demonstrates the benefit of knowledge gained in early functional biomarker studies. Neurocircuitry-related assessments remain a necessary and underutilized approach in drug development. Functional biomarker data can be used in the design of later-stage studies, including doses and objectives to be tested, leading to a more efficient drug-development process.

## Supplementary information

Supplemental Material
