# Supplementary material for: The D-amino acid oxidase inhibitor luvadaxistat improves mismatch negativity in patients with schizophrenia in a randomized trial
Source: Neuropsychopharmacology. 2023 Mar 16;48(7):1052–9. doi: 10.1038/s41386-023-01560-0 (PMC10018616; doi:10.1038/s41386-023-01560-0)
Supplement: Supplementary file 1 — Supplemental Material [file 41386_2023_1560_MOESM1_ESM.doc]

**Supplementary Materials and Methods**

**Eyeblink Conditioning.**

The procedure involved an initial set of eight unconditioned stimuli (US) (corneal air puff, 50 ms, 10 psi at source) presented with an intertrial interval of 15 s, which led to an unconditioned response (eyeblink). The acquisition phase then followed, consisting of five blocks of trials (mean intertrial interval: 15 s; range: 10 to 20 s). Each block contained 18 trials in which the conditioned stimulus (CS) (1 KHz tone; 400 ms; 80 dB sound pressure level) was paired with the US, and two trials in which the CS was presented alone. In trials in which the CS and US were paired, the US air puff was co-terminated with the CS tone. Learning is demonstrated when an eyeblink (the conditioned response) occurs in response to the CS (tone) before the onset of the US (air puff). Eyeblinks were measured with two bipolar electromyographic (EMG) electrodes (4 mm Ag/AgCl; SynAmp 2; Compumedics Neuroscan, Charlotte, NC, USA), one placed 1 cm below the left eyelid and centered with the pupil and the other placed 1 cm below the left lateral cantus. A ground electrode was placed on the forehead. EMG responses were continuously recorded (2.5 KHz A/D rate; high-pass filter = 1Hz; low-pass filter = 500 Hz; gain = 1000).

**Mismatch Negativity**

For MMN, participants were presented with 805 auditory stimuli, of which 644 (80%) were standard tones presented at 75 dB, 60 ms, 1000 Hz; 161 (20%) were duration-deviant tones at 75 dB, 150 ms, 1000 Hz. All tones had a 5 ms rise/fall time, with a stimulus onset asynchrony of 300 ms. A nose electrode served as reference. Eyeblink artifacts were minimized using a vertical electro-oculogram (VEOG)-based eyeblink spatial filter routine implemented in Neuroscan software. Records were then filtered at 0.1 to 30 Hz in 24 dB/octave, epoched (−50 to 300 ms), baseline-corrected, and threshold-filtered at ± 75 µV for artifact rejection, followed by visual inspection to exclude any overt artifacts from muscle contraction. Standard and deviant trials were averaged separately, followed by a subtraction of the two averaged waveforms. MMN was scored by peak detection within a 100 to 225 ms post-stimulus window by an automatic algorithm followed by visual inspection to verify correct placement of each marker for peak detection. Scoring was blinded.

**ASSR**

Seventy-five stimulus trains (trials) – each consisting of 15 clicks, with each click at 72 dB and of 1 ms duration – were delivered at each stimulus frequency. The duration ranged from 6 s per train for 2.5 Hz to 0.1875 s per train for 80 Hz, and the intertrain interval was 0.7 s. The six frequencies were presented in six separate blocks separated by 2 minutes. The order of the blocks was randomized.

The full-duration waveforms from each channel were epoched into 75 individual trials. Whereas typical ASSR analysis uses individual channels (often midline central electrode [CZ] or FZ), we adapted signal processing techniques [1] in which individual EEG channels are spatially combined to maximize response reliability using the denoising source separation (DSS) algorithm [2]. Raw ASSR power was calculated as the magnitude squared of the Fourier transform at the stimulus frequency. The Fourier transform was calculated using concatenated trials, rather than averaged trials, to increase spectral resolution [3]. Offline, signals were filtered at 0.8 Hz and detrended. Ocular artifacts were removed using the time-shift principal component analysis (PCA) algorithm, with ocular channels as references [4]. Normalized ASSR power at 40 Hz was then calculated as the mean over DSS components of the ratio of raw ASSR power and respective background power [3]. This combined use of DSS and normalized ASSR power represents critical improvements over previous ASSR power-extraction methods.

**Figure S1**

**CONSORT 2010 Flow Diagram**

**Allocation**

**Enrollment**

**Period 1**

**Period 2**

Assessed for eligibility (n= 53)

Excluded (n= 22)

♦  Not meeting inclusion criteria (n= 21 )

♦  Declined to participate (n= 0 )

♦  COVID-19 related shutdown (n=1 )

Received placebo
(n=5)

Assessed (n=5)

Allocated to 50 mg vs placebo (n=14)

Allocated to 500 mg vs placebo (n=17)

Randomized to sequence (n=31)

Received 500 mg
(n=8)

Assessed (n=8)

Received placebo
(n=9)

Assessed (n=9)

Received 50 mg
(n=7)

Assessed (n=7)

Received placebo
(n=7)

Assessed (n=7)

Lost to follow up
COVID-19 driven early termination (n=2)

Lost to follow up
(n=0)

Received 50 mg
(n=7)

Assessed (n=7)

Received placebo
(n=6)

Assessed (n=6)

Received 500 mg
(n=6)

Assessed (n=6)

Lost to follow up
voluntary withdrawal (n=2)

Lost to follow up
voluntary withdrawal (n=2)
protocol non-compliance (n=1)

**Figure S2**


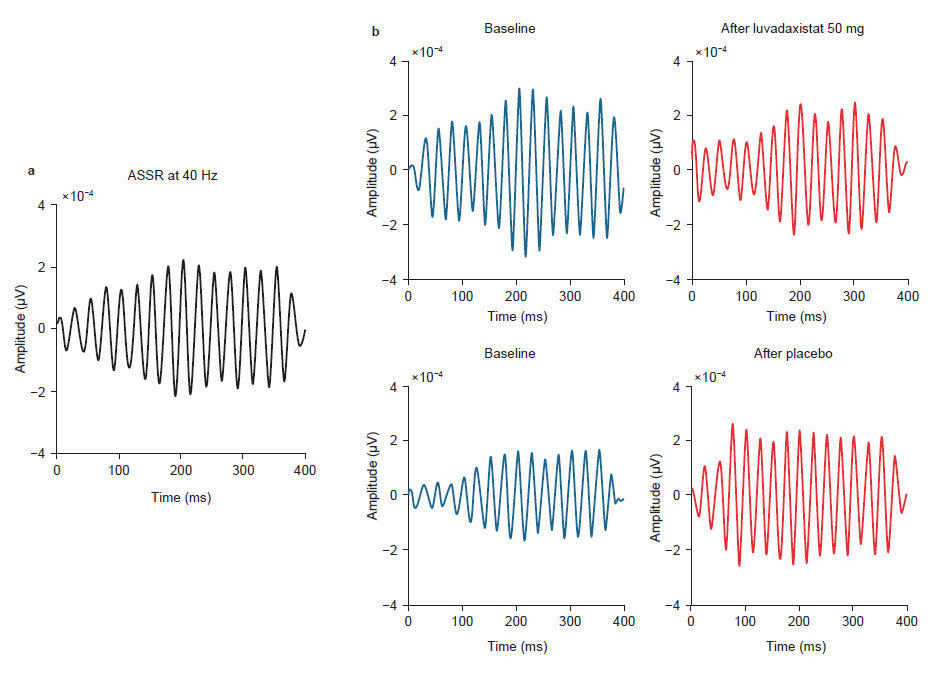


Waveforms of auditory steady state response at 40Hz from the cohort receiving 50 mg luvadaxistat or placebo. **a** Grand average of ASSR at 40Hz across all conditions. **b** Grand average of ASSR at baseline and following 50 mg luvadaxistat (top), and baseline and following placebo (bottom). These plots depict that the 40 Hz synchronizations were successfully generated in each condition; however, they do not represent the normalized power of 40 Hz ASSR used in data analysis, which was the ratio of the 40 Hz power to the power of its surrounding frequencies (e.g., 39 Hz and 41 Hz) (see Methods for more details).

**Figure S3**


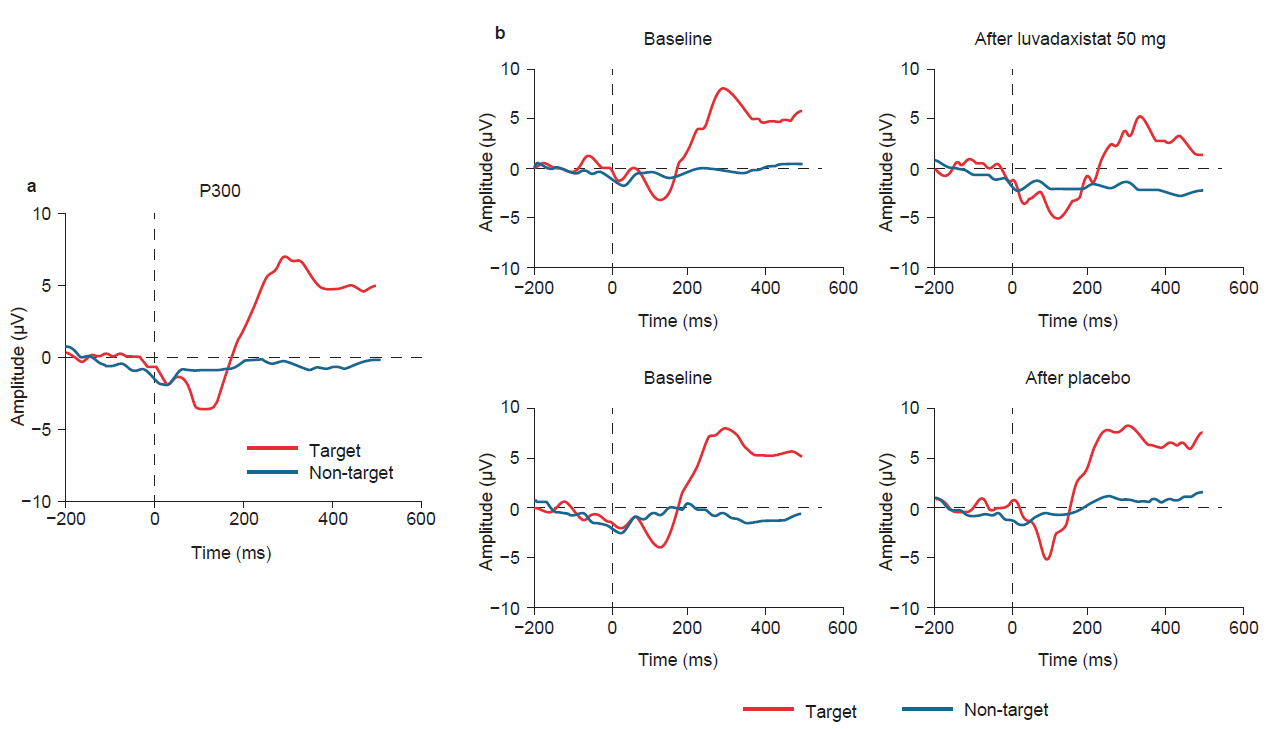


Waveforms of P300 from the cohort that received 50 mg luvadaxistat. **a** Grand average of P300 across conditions. Target (red) traces refer to the trials in which participants were asked to press the target button, and non-target (blue) traces are trials in which participants did not have to respond. **b** Grand averages of P300 at baseline and following 50 mg dosing (top), and at baseline and following placebo (bottom) for the same patients.

**Table S1**

**The Mean±SD values of D-serine, L-serine, D-serine/Total serine ratio and their corresponding percentage changes on each visit**

| **Dose**  **(mg)** | **Visit^1^** | **N** | **D-serine**  **(µg/mL)** | **%D-Serine Change** | **L-serine**  **(µg/mL)** | **%L-Serine Change** | **D-serine/total serine ratio** | **% Ratio change** |
| --- | --- | --- | --- | --- | --- | --- | --- | --- |
| 0 | 1 | 28 | 0.136±0.051 | 0 | 10.7±3.2 | 0.0 | 0.013±0.005 | 0 |
| 0 | 2 | 28 | 0.144±0.056 | 7.8±18.9 | 11.1±3.1 | 4.3±16.6 | 0.014±0.006 | 5.6±24.2 |
| 0 | 3 | 27 | 0.143±0.049 | 6.1±21.2 | 9.4±2.5 | -8.5±21.1 | 0.016±0.007 | 21.9±40.9 |
| 0 | 4 | 27 | 0.140±0.056 | 3.7±29.2 | 10.9±2.3 | 5.5±19.6 | 0.013±0.005 | 0.9±30.7 |
| 50 | 5 | 27 | 0.140±0.050 | 3.2±16.9 | 10.2±2.6 | -2.0±17.0 | 0.014±0.007 | 7.6±22.6 |
| 50 | 1 | 14 | 0.131±0.031 | 0 | 10.5±2.5 | 0.0 | 0.013±0.004 | 0 |
| 50 | 2 | 14 | 0.165±0.049 | 24.4±17.2 | 12.2±2.4 | 18.8±16.0 | 0.014±0.004 | 6.8±22.3 |
| 50 | 3 | 13 | 0.157±0.044 | 16.5±16.4 | 11.3±3.8 | 6.2±29.5 | 0.016±0.007 | 17.2±33.7 |
| 50 | 4 | 13 | 0.166±0.047 | 24.6±22.5 | 11.7±3.3 | 13.3±21.0 | 0.015±0.006 | 11.7±20.9 |
| 50 | 5 | 12 | 0.173±0.044 | 32.4±21.1 | 10.7±2.0 | 9.2±17.5 | 0.017±0.006 | 21.8±17.4 |
| 500 | 1 | 15 | 0.142±0.080 | 0 | 11.2±3.3 | 0 | 0.013±0.006 | 0 |
| 500 | 2 | 15 | 0.157±0.087 | 11.3±11.6 | 11.5±3.7 | 2.2±11.4 | 0.014±0.006 | 9.6±13.0 |
| 500 | 3 | 15 | 0.181±0.079 | 34.6±27.0 | 9.7±3.2 | -10.6+33.3 | 0.019±0.010 | 60.5±50.2 |
| 500 | 4 | 15 | 0.184±0.089 | 34.3±25.3 | 11.4±3.4 | 3.2±24.1 | 0.016±0.006 | 36.9±45.4 |
| 500 | 5 | 15 | 0.184±0.083 | 36.4±25.0 | 10.3±2.7 | -7.3±11.4 | 1.417±5.418 | 48.3±34.8 |

^1^ Visit 1 is baseline; visit 2 is Day 1, 3-6 h post first dose; visit 3 is Day 7 pre-dose, visit 4 is Day 7 3-6 h post-dose, visit 5 is Day 8 pre-dose.

**Table S2**

**Raw MMN amplitude values (µV, mean ± SEM)**

|  | 50 mg dose | |  | 500 mg dose | |
| --- | --- | --- | --- | --- | --- |
|  | Placebo | Luvadaxistat |  | Placebo | Luvadaxistat |
| Baseline | -1.411±0.410 | -0.795±0.136 |  | -1.260±0.283 | -1.547±0.286 |
| After treatment | -0.701±0.163 | -0.993±0.231 |  | -1.373±0.495 | -0.935±0.174 |

**REFERENCES**

1 Wang Y, Ding N, Ahmar N, Xiang J, Poeppel D, Simon JZ. Sensitivity to temporal modulation rate and spectral bandwidth in the human auditory system: MEG evidence. J Neurophysiol. 2012;107(8):2033-41.

2 de Cheveigne A, Parra LC. Joint decorrelation, a versatile tool for multichannel data analysis. Neuroimage. 2014;98:487-505.

3 Elhilali M, Xiang J, Shamma SA, Simon JZ. Interaction between attention and bottom-up saliency mediates the representation of foreground and background in an auditory scene. PLoS Biol. 2009;7(6):e1000129.

4 de Cheveigne A, Simon JZ. Denoising based on spatial filtering. J Neurosci Methods. 2008;171(2):331-9.
